# Morpho-Molecular Discordance and Cryptic Diversity in Jumping Bristletails: A Mitogenomic Analysis of *Pedetontus silvestrii* (Insecta: Archaeognatha: Machilidae)

**DOI:** 10.3390/insects16050452

**Published:** 2025-04-25

**Authors:** Wei Cen, Jia-Wen Li, Jia-Tao He, Xin-Yu Chen, Luo-Ying Li, Kenneth B. Storey, Dan-Na Yu, Jia-Yong Zhang

**Affiliations:** 1 College of Life Sciences, Zhejiang Normal University, Jinhua 321004, China; 2 Department of Biology, Carleton University, Ottawa, ON K1S 5B6, Canada; 3 Key Laboratory of Wildlife Biotechnology, Conservation and Utilization of Zhejiang Province, Zhejiang Normal University, Jinhua 321004, China

**Keywords:** Archaeognatha, mitochondrial genome, cryptic species, phylogenetic relationship, divergence time

## Abstract

Archaeognatha (bristletails) represent an evolutionarily significant but understudied insect group. Notably, the morphological identification method proposed by Mendes for Archaeognatha has certain limitations, which may lead to the underestimation or misidentification of some cryptic species. To address this issue, we employed an integrated strategy that combines morphological and molecular identification methods. Therefore, this study aimed to (1) identify cryptic diversity within *Pedetontus silvestrii* using mitogenomic data; (2) clarify phylogenetic relationships among Archaeognatha lineages; and (3) estimate divergence times for key taxonomic splits. We analyzed mitochondrial genomes from six *P. silvestrii* populations (Liaoning, Jilin, and Hebei Provinces) alongside 14 published Archaeognatha genomes. Key findings include the following: (1) Integrative analyses of genetic distances, phylogenetic reconstruction, bPTP-based molecular species delimitation, and divergence time estimation collectively revealed four evolutionarily distinct lineages within *P. silvestrii*. (2) Machilidae and Machilinae were non-monophyletic, whereas Petrobiellinae showed close affinity to Meinertellidae. (3) Archaeognatha originated ~301.19 Mya (Late Carboniferous); the Machilinae–Petrobiinae split occurred approximately 153.99 Mya (Jurassic). This study underscores the critical importance of mitogenomic analysis in elucidating cryptic biodiversity, while emphasizing the necessity of integrating morphological identification with molecular characterization for comprehensive species delineation in future taxonomic investigations.

## Simple Summary

Although there is morphological evidence suggesting the existence of cryptic diversity within Archaeognatha, unresolved challenges persist in defining species boundaries within this ancient insect lineage. To address this, we conducted a comprehensive mitochondrial genome analysis of six geographically isolated populations of the genus *Pedetontus*. By integrating mitochondrial genomic analysis, phylogenetic reconstruction, Bayesian Poisson Tree Processes (bPTP) analysis, and divergence time estimation, we identified four genetically distinct lineages, thereby confirming their status as cryptic species. Fossil-calibrated molecular dating further elucidated their speciation history.

## 1. Introduction

Bristletails (Insecta: Archaeognatha) are wingless insects characterized by several distinctive morphological features. The key morphological characteristics of Archaeognatha include contiguous compound eyes, a pair of ocelli, single-condyloid mandibles, seven-segmented maxillary palps, a central caudal filament, and two shorter lateral caudal filaments. Notably, Archaeognatha possess legs bearing coxal spines and abdominal segments equipped with spines and evertible vesicles [1,2]. Archaeognatha exhibit remarkable adaptability and inhabit a wide range of environments. They are globally distributed and typically reside in dark, moist microhabitats, including mosses, lichens, grasslands, forest leaf litter, tree bark, and decaying wood, and beneath stones and within soil. Additionally, they have been documented on coastal rocks. These nocturnal creatures feed on a diverse array of food sources, including algae, lichens, and mosses. During the day, they seek shelter under tree bark or within the leaf litter to avoid predators and harsh environmental conditions [3,4,5]. Archaeognatha, an ancient insect order of significant evolutionary interest, comprises approximately 500 species. This primitive order is systematically classified under two principal families: Machilidae (containing three taxonomically distinct subfamilies—Petrobiellinae, Petrobiinae, and Machilinae) and Meinertellidae [6].

Insect mitochondrial genomes generally preserve a conserved set of protein-coding genes, but exhibit structural rearrangements in specific lineages [7]. Despite the fact that the Archaeognatha mitogenome exhibits a gene arrangement nearly identical to that of *Drosophila yakuba* [8], the elevated mutation rates and reduced recombination frequencies of the former render them particularly valuable for resolving phylogenetic divergences [9,10,11,12,13]. The mitochondrial genome is characterized by limited recombination and complete linkage. Traditional phylogenetic analysis often relies on variations at a single locus to infer overall evolutionary relationships. However, if this locus is affected by selective pressure, abnormal mutation rates, or sequencing errors, this may lead to incorrect inferences of evolutionary branches. Therefore, phylogenetic analyses should integrate multiple gene loci or whole-genome data, and utilize high-throughput sequencing to improve the reliability of conclusions [14]. This progress is largely attributable to advancements in sequencing technology. Specifically, the widespread adoption of next-generation sequencing (NGS) has not only significantly reduced sequencing costs but also enabled efficient and precise sequencing of entire mitochondrial genomes [15,16].

Early taxonomic delineation of Archaeognatha prioritized ocular morphology, specifically quantifying two key morphometric parameters: the longitudinal-to-transverse proportion (L/W) and ommatidial separation index (CL/L). Additionally, other features utilized for classification include ocelli shape, antennal length, differences in sensory bristles, tibial length, and external genitalia characteristics [17,18,19]. The former classification system grouped Archaeognatha and Zygentoma under the order Thysanura [20]. Subsequent research led to their distinction and reclassification into separate orders: Archaeognatha and Zygentoma [21].

The phylogenetic relationships among families and genera within Archaeognatha remain a subject of significant controversy [22,23]. Current debates center on the evolutionary status of Machilidae, particularly regarding its potential paraphyly and inter-subfamilial evolutionary affiliations (Machilinae, Petrobiellinae, Petrobiinae) [2]. Early cladistic reassessment by Sturm and Bach [1] postulated the non-monophyletic nature of this family, resulting in the taxonomic exclusion of *Ditrigoniophthalmus*, *Charimachilis*, and *Mesomachilis*. Paleoentomological evidence from extinct Monura specimens, as demonstrated through tripartite fossil examination by Bechly and Stockar [24], positions this extinct order as the basal sister clade to modern Archaeognatha. While traditional morphological frameworks support Machilidae’s monophyly, contemporary phylogenomic investigations reveal significant incongruities challenging this long-standing classification. Ma et al. [5] constructed a phylogenetic tree by analyzing mitogenomes and demonstrated that Machilidae and Machilinae form paraphyletic groups. They also proposed that Petrobiellinae exhibits a closer phylogenetic relationship with Meinertellidae. Given that the internal phylogenetic relationships within Archaeognatha are not entirely consistent with morphology-based classifications, they suggested that the current taxonomic system may require revision. In a phylogenetic reconstruction of Archaeognatha, Zhang et al. [2] established an integrative methodological framework combining comparative morphology with multi-locus genomic markers; this was implemented through complementary statistical approaches: the maximum likelihood (ML) paradigm and Bayesian (BI) probabilistic modeling techniques. Their cladistic validation confirmed Meinertellidae as a monophyletic lineage and supported Machilidae as a paraphyletic group, aligning with the findings reported by Ma et al. [5]. However, the exclusion of the genera *Ditrigoniophthalmus* and *Petrobiellus* resulted in the remaining Machilidae taxa constituting a distinct clade. The monophyly of Machilinae and Petrobiinae subfamilies within Machilidae, however, remains unresolved given the currently available dataset [2].

Cryptic species, defined as morphologically similar yet genetically distinct taxa [25], represent a significant challenge in biodiversity research. Although traditional taxonomy has relied heavily on visual identification, species assessments have shifted from predominantly visually based morphological classification methods to integrated approaches combining morphological and molecular techniques, such as mitochondrial genome comparisons, *COI* gene sequence analyses, genetic distance calculations, and phylogenetic relationship evaluations [10,26,27].

In recent years, emerging methods such as molecular dating have found widespread application in the analysis of insect mitogenome data [28,29,30]. According to research by Shear et al. [31] and Labandeira et al. [32], certain fossil fragments recovered from Devonian deposits are considered to belong to Archaeognatha. These fragments are hypothesized to represent the earliest known fossil records of this order. Paleontological evidence documents three *Dasyleptus* specimens as the earliest confirmed archaeognathan fossils, dating to the Kasimovian–Gzhelian stages of the Late Carboniferous [33,34]. To date, researchers have identified a substantial number of relevant fossil species, preserved in amber, from diverse locations, including Lebanon, Myanmar, Mexico, and the Dominican Republic [34,35,36,37,38,39]. Despite the existing research, estimates of the divergence times within Archaeognatha remain inadequate. In their paleoentomological investigation, Zhang et al. [2] examined 15 Burmese amber-entombed archaeognathan specimens, implementing a combined-evidence analytical framework employing BI and maximum parsimony (MP) protocols with the integration of morphological characteristics and DNA markers. Phylogenetic reconstruction reveals an early divergence event between the Machilidae and Meinertellidae lineages that occurred significantly earlier than the Cretaceous period and was accompanied by a considerable evolutionary time span. Phylogenetic analyses revealed that the Machilidae–Meinertellidae split substantially predates the Cretaceous [2].

Based on the research of Zhang et al. [2], Montagna introduced two key fossil species, *Gigamachilis triassicus* and *Dasyleptus triassicus*, to reanalyze the phylogenetic relationships within Archaeognatha. Evolutionary analysis reveals that Machilidae emerged as early as the Middle Triassic (~240 Mya), establishing a divergence chronology that not only surpasses current paleontological evidence by ~200 Mya but also precedes the molecular data by a full geological epoch (100 Mya) [40]. Osozawa and Nel conducted molecular clock analysis using calibration points derived from fossil records of both the Quaternary and pre-Quaternary periods. Their results indicate that the crown group age of Insecta is estimated to be approximately 393.39 Mya, with Archaeognatha being considered the oldest lineage within Insecta [41].

It is worth noting that the type locality of *Pedetontus silvestrii* is the Korean Peninsula [18]. In this study, specimens were collected from six geographically distinct regions in northeastern China, adjacent to the Korean Peninsula: Gongchangling City, Dandong City, Xiuyan County, and Fengcheng City in Liaoning Province, China; Tonghua City in Jilin Province, China; and Chengde City in Hebei Province, China. This provides a foundation for testing the cryptic species hypothesis by considering geographical isolation as a potential driver of diversification. Morphological analysis reveals that the target samples exhibit a high consistency with the width-to-length ratio of the compound eyes of *Pedetontus silvestrii* (0.9–1.1) [18], making morphological differentiation challenging. Therefore, molecular-level investigations are warranted to complement the morphological studies. Although comprehensive cryptic species discovery ideally integrates behavioral, ecological, and phenological evidence alongside genetic data [42], this study focuses on assessing cryptic species in *Pedetontus silvestrii* through mitogenomic datasets, given the limited sample size, by employing a multi-evidence framework that incorporates four diagnostic approaches: morphological characteristics, genetic distance calculation, phylogenetic reconstruction, and intergenomic divergence time estimation. This integrative approach conclusively demonstrates the evolutionary distinctiveness indicative of cryptic speciation within this taxon.

## 2. Materials and Methods

### 2.1. Species Collection, Morphological Identification, and DNA Extraction

The samples for this study were collected from the following locations: Gongchangling (GCL) City, Liaoning Province, China (41°7′56″ N, 123°36′5″ E); Dandong (DD) City, Liaoning Province, China (41°0′26″ N, 124°21′16″ E); Xiuyan (XY) City, Liaoning Province, China (40°17′35″ N, 122°56′16″ E); Fengcheng (FC) City, Liaoning Province, China (40°27′9″ N, 124°4′4″ E); Tonghua (TH) City, Jilin Province, China (41°43′42″ N, 125°56′23″ E); and Chengde (CD) City, Hebei Province, China (40°57′9″ N, 117°57′46″ E). Six specimens were examined using an SMZ-1500 stereomicroscope (Nikon, Tokyo, Japan) for the morphological analysis. Based on diagnostic characteristics from Reference [18], these specimens were provisionally identified as *Pedetontus silvestrii*. Post-identification, specimens were cryopreserved in anhydrous ethanol at −20 °C within the Laboratory of Evolution and Molecular Ecology, Zhejiang Normal University, China. Muscle specimens underwent column-based nucleic acid isolation using the Ezup Column Animal Genomic DNA Purification Kit (Sangon Biotech, Shanghai, China), with strict adherence to the manufacturer’s protocols.

### 2.2. Mitogenome Sequencing and Assembly

Following DNA extraction, *COI* gene fragments were amplified with the universal insect primers LCO1490/HCO2198 [43], with subsequent species verification being performed through NCBI BLAST analysis (https://blast.ncbi.nlm.nih.gov/Blast.cgi, accessed on 2 December 2024). The mitochondrial genome was constructed using Sanger sequencing with 13 primer pairs from Zhang et al. [44]. All PCR products underwent electrophoresis purification prior to bidirectional sequencing (Sangon Biotech, Shanghai, China). Sequence assembly and validation were performed using DNASTAR v.6.0’s SeqMan Pro module [45].

### 2.3. Mitogenome Annotation and Sequencing Analysis

Mitochondrial annotation was performed using MITOS2 (Galaxy platform: https://usegalaxy.eu, accessed on 15 December 2024) for tRNA localization [46,47]. Comparative analysis of 12S/16S rRNA and 13 PCGs was conducted through Clustal W alignment in MEGA v.11 [48], supplemented by Kimura 2-parameter genetic distance calculations. PhyloSuite v.1.2.3 [49] enabled quantification of AT content and relative synonymous codon usage across six mitogenomes. Protein translation employed the invertebrate mitochondrial code [26,50,51]. Complete mitogenomes (GenBank: PV126555–PV126561) were visualized via CG View Server V1.0 (http://cgview.ca/, accessed on 20 January 2025) [52], with final graphical refinements accomplished in Adobe Illustrator 2024 [53]. Nucleotide composition biases were determined using standard skew formulas: AT skew = (A − T)/(A + T) and GC skew = (G − C)/(G + C) [54].

### 2.4. Phylogenetic Analyses

To explore the evolutionary relationships within Archaeognatha more comprehensively, we integrated the six mitogenomes generated in this study with fourteen additional Archaeognatha mitogenomes, and two mitogenomes of Collembola as outgroups, downloaded from NCBI (Table 1). The dataset included one species from Meinertellidae and nineteen species from Machilidae [5,6,44,55,56,57,58,59]. Two Collembola species, *Onychiurus orientalis* (NC_006074) and *Podura aquatica* (NC_006075), were selected as outgroups [60]. The 13 PCGs were processed through an integrated phylogenetic pipeline in PhyloSuite v1.2.3 [49]: (1) MAFFT-based alignment [61], (2) Gblocks v0.91b filtering of hypervariable regions [62], and (3) sequence concatenation using built-in tools. Phylogenetic reconstruction employed both BI and ML, using all three codon positions from the 13 PCGs nucleotide sequences. [63]. This conservative approach followed codon saturation detection in DAMBE v.7.3.11 [64], in which the third codon positions did not exhibit substitution saturation. Evolutionary model selection was performed through PartitionFinder v2.2.1 (BIC criterion) [65], with the optimal partitioning schemes and substitution models documented in Table 2. Phylogenetic reconstruction combined (1) ML analysis via IQ-TREE v2.0 (1000 ultrafast bootstraps) [66] and (2) BI implementation in MrBayes v3.2 (10M MCMC generations, 25% burn-in) [67]. Final tree visualization was employed in FigTree v.1.4 [68] and was further refined for presentation using Adobe Illustrator [53]. Following phylogenetic reconstruction, we performed post hoc species delimitation using the bPTP (Bayesian Poisson Tree Processes) model [69] to assess whether the lineage splits were statistically supported. Input files were prepared by (1) exporting the finalized ML tree (Newick format) from IQ-TREE and (2) focusing specifically on six *P. silvestrii* specimens. The analysis was executed on the bPTP server (http://species.h-its.org/ptp/, accessed on 24 February 2025), with the following default parameters: 100,000 MCMC generations, thinning = 100, burn-in = 10%, and convergence assessed via ESS values >200. Species clusters were considered well supported if their posterior probabilities were ≥0.95.

### 2.5. Divergence Time Estimation

Fossils of Archaeognatha are exceptionally rare, and the calibration points derived from reported fossils remain ambiguous. Fossil calibration points are essential for estimating divergence times [70,71,72]. Fossil records were retrieved from the online platform (https://paleobiodb.org/classic, accessed on 15 January 2025) to obtain relatively accurate temporal data. According to Misof et al. [73], who constructed an MCMC tree for Insecta, the divergence time between Archaeognatha and Collembola is estimated to be approximately 479 million years ago (Mya), corresponding to the Early Ordovician period. We adopted this estimate as the root age for our analyses. We selected four fossil calibration points from the literature: (1) The oldest known fossils of Archaeognatha include *D. lucasi* Brongniart, 1885; *D. noli* Rasnitsyn, 1999; and *D. rowlandi* Rasnitsyn, 2004. These fossils provide reliable calibration points for Archaeognatha and are dated to approximately 298.9 to 303.7 million years ago (Mya) [33,34,74]. (2) The secondary calibration derives from *Machilis acuminata* fossil evidence (Paleogene: 33.9–38.0 Mya), establishing robust chronological constraints for the genus [75]. (3) The third calibration derives from *Gigamachilis triassicus*, dated to the Triassic period, between 235 and 242 Mya, and serving as a calibration for the family Machilidae [76]. (4) The final calibration derives *Cretaceomachilis libanensis* from the family Meinertellidae, an event dated to the Early Cretaceous period, approximately 125.45 to 130.00 Mya [35].

Divergence time estimation for Archaeognatha was conducted in MCMCTree (PAML v4.8) [77] using the ML tree topology. The analysis parameters included the GTR evolutionary model with constraint RootAge < 4.79 (usedata = 3). The Markov chain Monte Carlo configuration comprised 1×10^6^ burn-in generations followed by 10^4^ sample collections at 1000-generation intervals. To ensure adequate mixing of the MCMC chain, we utilized Tracer v1.7.1 [78] to verify that the effective sample sizes (ESS) for all parameters were consistently above 200 [79]. Finally, the divergence times of each branch were visualized using FigTree v1.4 [68] and refined with Adobe Illustrator [53].

## 3. Results

### 3.1. Composition of Mitogenomes

All six mitogenomes comprised double-stranded DNA molecules, with lengths varying from 15,705 bp for *Pedetontus silvestrii* GCL to 15,872 bp for *P. silvestrii* FC (Table S1). The six *P. silvestrii* mitogenomes exhibited the typical insect gene arrangement, consisting of 37 standard genes (13 PCGs, 22 tRNAs, 2 rRNAs) with strand-specific distribution patterns (H-strand: 9 PCGs + 14 tRNAs; L-strand: 4 PCGs + 8 tRNAs + 2 rRNAs). These genomes showed characteristic AT-rich composition (72.8–75.2% overall) and consistent strand asymmetry (positive AT-skew, negative GC-skew). Key features included the following: (1) ATN start codons and TAA termination predominated (with incomplete stops in *COI-III* and *ND5*) (Table S2). (2) The four most frequent codons were UUU (Phe), UUA (Leu), AUU (Ile), and AUA (Met), each exceeding 200 occurrences (Figure S3; Tables S3–S8). (3) The conserved rRNA positions were (16S: trnL-trnV; 12S: trnV-CR) with 75.4–77% AT-content. (4) The control region was located between 12S rRNA and trnI.

### 3.2. Estimation and Evaluation of Genetic Distance

Based on the compositional differences in mitogenomes, genetic distances were calculated using MEGA software. We analyzed the complete mitogenomes of seven sequences: *P. silvestrii* DD, *P. silvestrii* FC, *P. silvestrii* GCL, *P. silvestrii* TH, *P. silvestrii* CD, *P. silvestrii* XY, and the reference sequence of *P. silvestrii* from the NCBI (accession number NC_011717). The results are summarized in Table 3. The genetic distances among all species ranged from 1.64% to 19.54%, with an average of 14.80%. Specifically, the genetic distance between *P. silvestrii* DD and *P. silvestrii* FC was 1.64%, between *P. silvestrii* DD and *P. silvestrii* XY was 6.74%, and between *P. silvestrii* FC and *P. silvestrii* XY was 6.72%. Furthermore, the genetic distances between the six *P. silvestrii* strains (DD, XY, FC, GCL, TH, and CD) and the reference sequence (NC_011717) were as follows: 18.48% for DD, 18.22% for FC, 19.16% for XY, 18.48% for GCL, 19.54% for TH, and 10.69% for CD. All of these distances exceeded 10%. It is noteworthy that the genetic distances among *P. silvestrii* exhibit a consistent pattern. Specifically, the genetic distances between *P. silvestrii* GCL and the other five strains (DD, FC, TH, XY, CD) ranged from 11.70% to 18.08%, with precise values of 17.21% for DD, 17.10% for FC, 17.83% for TH, 11.70% for XY, and 18.08% for CD. Additionally, the genetic distances between *P. silvestrii* TH and the three strains (DD, FC, XY) were notably close, at 11.78% for DD, 11.48% for FC, and 11.65% for XY. Furthermore, the genetic distances between *P. silvestrii* CD and the other five strains (DD, FC, GCL, TH, XY) ranged from 10.69% to 19.41%, with specific values of 18.27% for DD, 17.94% for FC, 18.08% for GCL, 19.41% for TH, and 18.71% for XY.

To validate this conclusion, we conducted additional analyses using MEGA to calculate genetic distances both within and between groups. Group 1 included the three samples identified as the same species (*P. silvestrii* DD, *P. silvestrii* FC, and *P. silvestrii* XY), whereas Groups 2 to 5 comprised *P. silvestrii* GCL (Group 2), *P. silvestrii* TH (Group 3), *P. silvestrii* CD (Group 4), and *P. silvestrii* (NC_011717) (Group 5), respectively. The genetic distances between these groups are detailed in Table S9. The results indicate that inter-group genetic distances exceeded 10%, whereas the intra-group genetic distances within Group 1 were 5%.

Based on genetic distance analyses (including comparisons among samples, within groups and between groups), the six *P. silvestrii* specimens examined in this study are grouped into four distinct lineages, designated as Clades A, B, C, and D. Clade A includes *P. silvestrii* DD, FC, and XY, forming a sister branch ((*P. silvestrii* DD + FC) + XY) with *P. silvestrii* TH. Clade B is exclusively represented by *P. silvestrii* TH. Since *P. silvestrii* GCL forms a sister branch to (((*P. silvestrii* DD + FC) + XY) + TH), it is classified as Clade C. Furthermore, given that *P. silvestrii* CD occupies a distinct branch separate from the other five specimens, it is assigned to Clade D.

### 3.3. Phylogenetic Relationship

**Figure 1:**
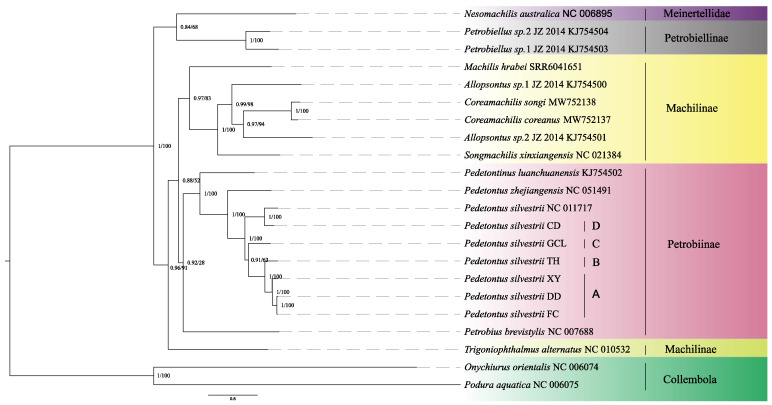
Evolutionary reconstruction employing 13 protein-coding genes was conducted across 22 mitochondrial genomes, including 6 novel assemblies generated in this investigation and 16 genomic references retrieved from public databases. Nodal support indices are annotated as Bayesian posterior probabilities (**left**) and maximum-likelihood bootstrap supports (**right**). Specifically, the cryptic species lineages are as follows: A represents the XY/DD/FC lineage of *P. silvestrii*, B represents the TH lineage of *P. silvestrii*, C represents the GCL lineage of *P. silvestrii*, and D represents the CD lineage of *P. silvestrii*.

Phylogenetic reconstructions employing Bayesian and maximum-likelihood methodologies demonstrated congruent topological arrangements (Figure 1). Phylogenetic analyses supported the monophyly of Archaeognatha while indicating paraphyly within Machilidae and Machilinae. Additionally, the results revealed a close phylogenetic relationship between Petrobiellinae and Meinertellidae. Given the restricted taxonomic sampling of the Meinertellidae species included in this study, additional investigations are necessary to verify the monophyletic status of this family.

Both the BI and ML phylogenetic trees indicate that *P. silvestrii* DD and *P. silvestrii* FC form a sister clade, which together with *P. silvestrii* XY constitutes a larger clade represented as ((*P. silvestrii* DD + *P. silvestrii* FC) + *P. silvestrii* XY). Furthermore, both trees suggest the relationship of (((*P. silvestrii* DD + *P. silvestrii* FC) + *P. silvestrii* XY) + *P. silvestrii* TH) with a high prior probability and bootstrap values. Additionally, *P. silvestrii* CD and *P. silvestrii* (NC_011717) are sister groups. The branch (((*P. silvestrii* DD + *P. silvestrii* FC) + *P. silvestrii* XY) + *P. silvestrii* TH) + *P. silvestrii* GCL) is also supported as a sister clade to (*P. silvestrii* CD + *P. silvestrii* (NC_011717)). The bPTP analysis confirmed the distinctness of *P. silvestrii* lineages, with posterior probabilities exceeding the significance threshold (Figure S2). However, no significant divergence was detected between *P. silvestrii* DD and *P. silvestrii* FC (posterior probability = 0.04, below the significance threshold of 0.05), suggesting conspecificity. In contrast, all other comparisons strongly supported independent species status, aligning with phylogenetic divergence patterns.

### 3.4. Estimation of Divergence Time

**Figure 2:**
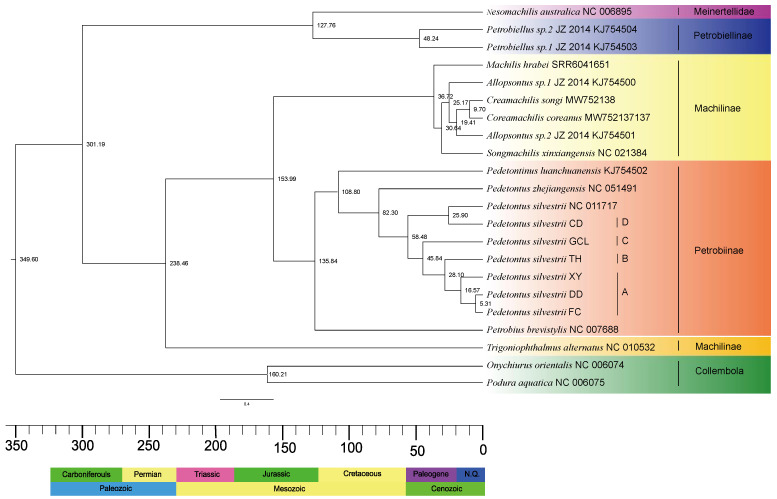
The evolutionary timescale of Archaeognatha was inferred from protein-coding gene (PCG) data using four fossil calibration points. The median age estimates for each node are displayed above their respective nodes. The stratigraphic chronology is displayed along the lower margin of the illustration. Specifically, the cryptic species lineages are as follows: A represents the XY/DD/FC lineage of *P. silvestrii*, B represents the TH lineage of *P. silvestrii*, C represents the GCL lineage of *P. silvestrii*, and D represents the CD lineage of *P. silvestrii*.

We utilized an ML/BI tree topology based on 13 protein-coding genes (PCGs) for divergence time estimation. The results (Figure 2) indicate that Archaeognatha diverged in the Late Carboniferous Period [301.19 Mya; 95% highest posterior density (HPD): 298.88–303.68 Mya]. Machilidae originated in the Middle Triassic [238.46 Mya; 95% HPD: 235.01–241.99 Mya], and the most recent common ancestor (MRCA) of Meinertellidae and Petrobiellinae emerged in the Early Cretaceous [127.76 Mya; 95% HPD: 125.45–130.00 Mya]. The MRCA of Machilinae and Petrobiinae diverged during the Late Jurassic Period [153.99 Mya; 95% HPD: 89.91–227.67 Mya]. It is worth noting that Petrobiinae diverged in the Early Cretaceous [135.84 Mya; 95% HPD: 76.95–212.73 Mya], and Petrobiellinae approximately during the Eocene Epoch [48.24 Mya; 95% HPD: 15.89–101.83 Mya]. Additionally, the estimated genus-level divergence times are as follows: *Machilis* in the the Eocene period [36.72 Mya; 95% HPD: 34.19–38.10 Mya], *Coreamachilis* during the Miocene Epoch [19.41 Mya; 95% HPD: 12.98–25.76 Mya], *Songmachilis* during the Oligocene Epoch [30.64 Mya; 95% HPD: 24.80–34.98 Mya], *Pedetontinus* in the Early Cretaceous [108.80 Mya; 95% HPD: 60.31–179.64 Mya], and *Pedetontus* during the Late Cretaceous [82.30 Mya; 95% HPD: 41.11–146.60 Mya]. Our findings suggest that the genera *Allopsontus*, *Machilis*, *Coreamachilis*, and *Songmachilis* exhibited similar divergence times during the Cenozoic Era. The MRCA of the six species sequenced in this study and *P. silvestrii* (NC_011717) was estimated to have diverged approximately in the Eocene Epoch [58.48 Mya; 95% HPD: 30.01–105.76 Mya]. Following the separation of *P. silvestrii* CD, *P. silvestrii* GCL diverged during the Eocene Epoch [45.84 Mya; 95% HPD: 23.20–87.97 Mya]. *P. silvestrii* TH and *P. silvestrii* XY separated from their respective branches in the Oligocene Epoch [28.10 Mya; 95% HPD: 12.93–56.90 Mya] and Pleistocene Epoch [16.57 Mya; 95% HPD: 6.97–32.78 Mya], respectively. Our analysis indicates that the divergence between *P. silvestrii* DD and *P. silvestrii* FC occurred during the Pleistocene Epoch [5.31 Mya; 95% HPD: 1.41–13.92 Mya]. The divergence times of key nodes and branches are summarized in Table 4.

## 4. Discussion

### 4.1. Comparison of Mitogenome Composition

Our sequencing of six complete *P. silvestrii* mitogenomes reveals remarkable genomic conservation within this lineage. The observed structural features, including universal ATN initiation codons and characteristic truncated termination in *COI-III* and *ND5*, further substantiate the high level of conservation of the mitochondrial genome [80]. The dynamic interplay among mutational processes, selective constraints, and stochastic genetic drift serves as a key determinant of codon usage bias, thereby underscoring the crucial role of codon usage analyses in elucidating molecular evolutionary patterns within genomes [81,82]. Codon selection is influenced by the adenine–thymine mutation bias [83,84], leading to a reduced utilization of codons with G or C at the third position, as shown in Tables S3–S8. The extreme conservation of *P. silvestrii* mitogenomes suggests they may serve as reliable markers for phylogenetic studies within Archaeognatha, minimizing concerns about structural homoplasy. The limited codon usage variation among specimens reinforces the value of mitochondrial sequences for population-level analyses in this taxon.

### 4.2. Phylogenetic Relationship and Divergence Time of Archaeognatha

The phylogenetic tree generated in this study strongly supports the monophyly of Archaeognatha, while revealing paraphyly in both Machilidae and Machilinae. Petrobiellinae is shown to be closely related to Meinertellidae. These findings are consistent with previous studies by Ma et al. and Guan et al. [5,59]. However, the limited taxonomic sampling for Meinertellidae, represented by only one species, restricts our ability to robustly evaluate its phylogenetic relationships. Although our research results have contributed to the validation of previous studies on the phylogenetics of Archaeognatha, it must be acknowledged that there are some key limitations. Our phylogenetic reconstruction employed standard maximum-likelihood and Bayesian approaches, consistent with previous studies in this group [5].

Through a novel molecular dating approach combining (1) five carefully selected fossil calibrations (four node calibrations + one root age) from a comprehensive review of the literature and the Fossil Calibration Database, and (2) MCMCTree (PAML v4.8) with autocorrelated relaxed-clock modeling, we obtained high-resolution divergence estimates for Archaeognatha evolution. Our present results reveal that the internal diversification of Archaeognatha occurred in the Late Carboniferous (approximately 301.19 Mya), whereas Machilidae underwent diversification later during the Middle Triassic (approximately 238.46 Mya). These temporal patterns strongly corroborate the findings of Sturm and Bach [1], confirming that the divergence between Machilidae and Meinertellidae predates the Cretaceous period, with Machilidae exhibiting an earlier evolutionary origin than Meinertellidae. Furthermore, phylogenetic reconstruction reveals that Meinertellidae diverged from other Archaeognatha species in the Early Cretaceous (approximately 127.76 Mya), a finding that directly supports the evolutionary hypothesis formulated by Sturm and Poinar [35]. Through divergence time estimation, we determined that the genus *Machilis* originated approximately 36.72 Mya, which exhibits remarkable congruence with the Eocene-origin framework (32–56.5 Mya) proposed by Zhang et al. [2].

Archaeognatha is a herbivorous insect that primarily feeds on spore-producing plants such as bryophytes and lichens [85]. Its evolutionary trajectory correlates with angiosperm biomass dynamics, as evidenced by preserved paleobotanical assemblages from the Late Pennsylvanian (299–307 Mya) which indicate that spore-producing plants were predominant during this period [86]. In the present study, we estimated that the internal diversification of Archaeognatha occurred approximately 301.19 Mya, coinciding with the prevalence of spore-producing plants in the Late Pennsylvanian [87]. This temporal alignment supports the hypothesis that the dietary preferences of Archaeognatha were consistent with the dominant vegetation of the period. Notably, the Late Carboniferous was characterized by a cold phase in geological history, which was marked by the expansion and contraction of glaciers. These glacial cycles led to significant climatic fluctuations and changes in vegetation types. During this period, these environmental shifts likely influenced terrestrial ecosystems, driving adaptive changes in terrestrial animals [88,89]. We hypothesize that these environmental factors may have contributed to the diversification of Archaeognatha and the emergence of new species during this period.

The Mesozoic Era witnessed substantial transformations in global climate and vegetation compared to the Paleozoic Era, with particularly marked ecological shifts occurring during the Cretaceous period [90,91]. Fossil evidence indicates that the earliest angiosperms appeared in the Early Cretaceous [92]. The diversification of epiphytic lichens is closely linked to the varied characteristics of angiosperm bark, such as pH levels, water retention capacity, and hardness, which provided a range of ecological niches and promoted adaptive radiation in lichens [93]. Additionally, the initial radiation of angiosperms created new ecological opportunities for bryophytes, driving their diversification [94]. The subsequent diversification of bryophytes and lichens facilitated the differentiations of the Archaeognatha species that primarily feed on these groups. Crucially, our phylogenetic analyses reveal that a large portion of extant Archaeognatha lineages originated following the Cretaceous–Paleogene (K-Pg) boundary (65.5 Mya), suggesting that the proliferation of angiosperms laid the foundation for the diversification of Archaeognatha. This temporal congruence highlights the potential co-evolutionary relationship between angiosperms and Archaeognatha, underscoring the importance of plant–insect interactions in shaping biodiversity.

The Neogene glacial events were characterized by prolonged durations and low atmospheric carbon dioxide concentrations, which triggered significant biogeographic differentiation [95]. These effects promoted stable evolution and a gradual increase in biodiversity. Against this background, the diversification of bryophytes and lichens reached a peak during the Miocene–Pliocene Epochs [96,97]. This study reveals that the divergence times of the four cryptic species lineages are significantly correlated with Neogene glacial periods. Lineage A diverged during the active glacial phases of the Neogene, whereas the other three lineages corresponded to glacial events during the Paleogene. Our findings demonstrate that Neogene glacial–interglacial oscillations acted as a key driver of morphologically cryptic diversification, which was facilitated by sustained habitat partitioning and competitive exclusion mechanisms.

### 4.3. Identification of Cryptic Species

Utilizing Mendes’ description of *P. silvestrii* [18], we found that the six samples involved in this study exhibited no significant morphological differences and all were identified as *P. silvestrii*. However, we noticed that the ratio of the width to the length of the compound eyes of these samples fluctuated between 0.9 and 1.1, suggesting the potential presence of cryptic species within *P. silvestrii*. Therefore, it is essential to conduct further in-depth investigations from both morphological and molecular biological perspectives to confirm this hypothesis.

The genetic divergences between the A lineage of *P. silvestrii* and the B, C, and D lineages were 17.3%, 11.6%, and 18.3%, respectively (Table S9), all exceeding 10%. Phylogenetic analysis revealed that the A lineage is a sister group to the B lineage, but forms a distinct clade separate from the C and D lineages (Figure 1). The A lineage diverged approximately 16.57 Mya [95% HPD: 6.37–32.78 Mya], which is significantly later than the divergence of the B lineage at 28.10 Mya [95% HPD: 12.93–56.90 Mya], with a time difference exceeding 10 Mya (Figure 2). Similarly, the genetic divergences between the B lineage and the C and D lineages also exceeded 10% (Table S9). Both the ML and BI phylogenetic trees indicate that the B clade forms an evolutionary clade different from the C and D clades (Figure 1). The B lineage diverged more recently compared to the C lineage, which diverged approximately 45.84 Mya [95% HPD: 23.20–87.97 Mya], with a divergence time difference exceeding 10 Mya (Figure 2). Additionally, the genetic distance between the C and D lineages exceeded 10% (Table S9), and both the ML and BI phylogenetic analyses provide strong evidence that the C and D lineages constitute distinct evolutionary lineages (Figure 1). The C lineage diverged later than the D lineage, which originated approximately 58.48 Mya [95% HPD: 26.92–98.20 Mya], with a divergence time gap exceeding 10 Mya (Figure 2). To conclude, our results demonstrate that the genetic distances among the four lineages A, B, C, and D are significantly higher than the intraspecific differentiation threshold (typically < 2%) and approach interspecific levels (>10%). Furthermore, divergence time differences between these lineages all exceed 10 Mya. According to the dual criteria for identifying cryptic species, which encompass genetic differentiation exceeding 10% and divergence time differences among species greater than 10 Mya [9,26], this study confirms that these lineages represent four distinct evolutionary units within *P. silvestrii*.

Analysis using the Bayesian molecular species delimitation software bPTP revealed that the highest Bayesian-supported solution between *P. silvestrii* DD and *P. silvestrii* FC was 0.04, meeting the criteria for conspecificity [69]. By contrast, the highest Bayesian-supported solutions between other *P. silvestrii* samples and this sister clade were significantly higher than this threshold, indicating that they represent species distinct from *P. silvestrii* DD/FC. Based on these findings, this study verified the conclusion that *P. silvestrii* DD and *P. silvestrii* FC belong to the same cryptic species lineage.

Speciation results from the interplay of geographic isolation, ecological divergence, and reduced gene flow [42,98,99]. In our study, mountains and rivers have isolated *Pedetontus* populations across Northeast China (six sites in Liaoning, Jilin, and Hebei), in areas separated from the Korean Peninsula type locality. Three Liaoning populations (*P. silvestrii* XY, DD, FC) occur within 100 km, while others show greater isolation (>200 km). Combined with Archaeognatha’s limited dispersal capacity, this geographic pattern explains the four distinct mitochondrial lineages through long-term isolation, reducing gene flow; local adaptation; and genetic drift accumulation. While our study demonstrates significant mitochondrial divergence and geographic isolation in *Pedetontus* lineages, we acknowledge that behavioral and fine-scale ecological factors could further reinforce cryptic speciation. These aspects were not examined here due to the technical challenges of maintaining live Archaeognatha populations for controlled experiments.

In conclusion, our analyses have identified four distinct evolutionary lineages within *P. silvestrii*, each corresponding to a cryptic species. These findings are supported by genetic distances, phylogenetic relationships, and divergence times. Based on our results, we speculate that cryptic species may be prevalent within the genus *Pedetontus*. However, due to the limited sample size, further identification of these potential cryptic species remains a challenge. Future research will aim to address this issue to provide a more comprehensive understanding.

## 5. Conclusions

In this study, we sequenced the complete mitogenomes of six specimens of *P. silvestrii*, thereby enhancing our understanding of the mitogenomes within the genus *Pedetontus*, especially with reference to *P. silvestrii*, and validated the phylogenetic analyses of Archaeognatha conducted by our predecessors. Mitogenomes have been widely utilized in molecular phylogenetics due to their numerous advantages, including the ability to construct robust phylogenetic trees with comprehensive taxonomic sampling. Through genetic distance analysis at both the species and the group level, phylogenetic tree construction, molecular species delimitation using the bPTP server, and divergence time estimation, this study identified four cryptic species, namely, *P. silvestrii* DD/XY/FC, *P. silvestrii* GCL, *P. silvestrii* TH, and *P. silvestrii* CD. Using four reliable fossil calibration points, this study supports the conclusion that the diversification of Archaeognatha began approximately 301.19 Mya, with significant diversification occurring during the Paleogene Epoch. This study establishes a robust phylogenetic framework for Archaeognatha, integrating molecular datasets with crucial paleontological evidence to elucidate early divergence events within insect evolution.

## Figures and Tables

**Table 1:** Taxonomic hierarchy, scientific nomenclature, mitogenome sizes, and corresponding NCBI accession codes are systematically documented for all 22 taxa investigated.

Family	Subfamily	Species	Length (bp)	GenBank Accession Number
Meinertellidae	/	*Nesomachilis australica*	15,474 bp	NC_006895
Machilidae	Petrobiellinae	*Petrobiellus* sp. 1 JZ-2014	15,843 bp	KJ754503
		*Petrobiellus* sp. 2 JZ-2014	14,022 bp	KJ754504
	Machilinae	*Allopsontus* sp. 1 JZ-2014	15,532 bp	KJ754500
		*Allopsontus* sp. 2 JZ-2014	15,538 bp	KJ754501
		*Trigoniophthalmus alternatus*	16,197 bp	NC_010532
		*Machilis hrabei*	15,585 bp	PV126557
		*Coreamachilis coreanus*	15,578 bp	MW752137
		*Coreamachilis songi*	15,570 bp	MW752138
		*Songmachilis xinxiangensis*	15,473 bp	NC_021384
	Petrobiinae	*Petrobius brevistylis*	15,698 bp	NC_007688
		*Pedetontinus luanchuanensis*	14,106 bp	KJ754502
		*Pedetontus zhejiangensis*	15,602 bp	NC_051491
		*Pedetontus silvestrii*	15,879 bp	NC_011717
		*Pedetontus silvestrii* DD	15,742 bp	PV126559
		*Pedetontus silvestrii* FC	15,872 bp	PV126560
		*Pedetontus silvestrii* GCL	15,705 bp	PV126561
		*Pedetontus silvestrii* TH	15,770 bp	PV126555
		*Pedetontus silvestrii* CD	15,747 bp	PV126558
		*Pedetontus silvestrii* XY	15,749 bp	PV126556
Collembola		*Onychiurus orientalis*	12,984 bp	NC_006074
		*Podura aquatica*	13,809 bp	NC_006075

**Table 2:** The optimal model and partitioning scheme selected for the phylogenetic analysis, along with the model name and corresponding number of sites for each partition.

Best Model	Sites	Partition Names
GTR + I + G	1181	ND3_codon1, ATP6_codon1, COIII_codon1, COII_codon1, Cyt b_codon1
GTR + I + G	1680	COI_codon2, ND3_codon2, ATP6_codon2, COII_codon2, COIII_codon2, Cyt b_codon2
TVM + G	1728	ND3_codon3, COII_codon3, ATP8_codon3, ATP6_codon3, COI_codon3, COIII_codon3, Cyt b_codon3
GTR + I + G	528	ATP8_codon2, ATP8_codon1, ND2_codon1, ND6_codon1
SYM + I + G	499	COI_codon1
GTR + I + G	1327	ND1_codon1, ND5_codon1, ND4L_codon1, ND4_codon1
GTR + I + G	1327	ND4_codon2, ND5_codon2, ND4L_codon2, ND1_codon2
HKY + G	1327	ND4L_codon3, ND5_codon3, ND4_codon3, ND1_codon3
TVM + G	432	ND2_codon2, ND6_codon2
HKY + G	432	ND2_codon3, ND6_codon3

**Table 3:** The genetic distances among the seven mitochondrial genomes of *P. silvestrii*, comprising *P. silvestrii* DD, FC, XY, GCL, TH, CD, and the reference sequence *P. silvestrii* (GenBank accession: NC_011717).

	CD	DD	FC	XY	GCL	TH
*P. silvestrii* CD						
*P. silvestrii* DD	0.1827					
*P. silvestrii* FC	0.1794	0.0164				
*P. silvestrii* XY	0.1871	0.0674	0.0672			
*P. silvestrii* GCL	0.1808	0.1721	0.1710	0.1170		
*P. silvestrii* TH	0.1941	0.1178	0.1148	0.1165	0.1783	
*P. silvestrii* NC_011717	0.1069	0.1848	0.1822	0.1916	0.1848	0.1954

**Table 4:** Mitogenome-inferred node/branch ages across Archaeognatha are calibrated in Mya, with ampersand (&) symbology defining phylogenetic associations.

Nodes/Clades	Mean Divergence Time (Mya)	95% HPD Range (Mya)
Meinertellidae	127.76	125.45, 130.00
Machilidae	238.46	235.01, 241.99
Meinertellidae & Machilidae	301.19	298.88, 303.68
Meinertellidae & Petrobiellinae	48.24	15.89, 101.83
Machilinae & Petrobiinae	153.99	89.91, 227.67

## Data Availability

Data used to support this study are available from the National Center for Biotechnology Information (https://www.ncbi.nlm.nih.gov) (accessed on 20 December 2024). The GenBank numbers are PP577161-PP577163.

## Supplementary Materials

The following supporting information can be downloaded at: https://www.mdpi.com/article/10.3390/insects16050452/s1, Figure S1. Circular genome maps illustrate the complete mitochondrial genomes of six *P. silvestrii* variants: DD, FC, GCL, TH, CD, and XY. Strand-specific coding is represented through concentric circles, with the outer circle (positive strand/5′→3′) contrasting with the inner circle (negative strand/3′→5′). Subsequent rings depict (1) GC-skew deviations, and (2) variations in GC content relative to genomic averages. Figure S2. The results from the Bayesian phylogenetic species delimitation analysis (bPTP) server. The bPTP analysis determines the number of different species by evaluating genetic differences and phylogenetic relationships. This figure includes the posterior probabilities supporting species boundaries and the hierarchical structure of species clusters. Figure S3. Synonymous codon usage bias in the *Pedetontus silvestrii* mitogenome was systematically evaluated through codon preference analysis. The visualization framework employs codon variants mapped along the abscissa and RSCU measurements plotted on the ordinate. This metric quantifies codon preference by measuring observed-to-expected usage ratios across translational coding sequences. Table S1. Comprehensive data on the base composition of the mitochondrial genomes of *Pedetontus silvestrii* variants. This table includes metrics such as base length (total number of bases), AT content (percentage of adenine and thymine), AT skew ([(A − T)/(A + T)]), and GC skew ([(G − C)/(G + C)]). The data are presented for various genomic regions, facilitating a detailed examination of compositional biases. Table S2. A comprehensive annotation of the mitochondrial genomes for *Pedetontus silvestrii* variants. This details the positions and start and stop codons, as well as other relevant features, for each gene, including protein-coding genes, tRNA genes, and rRNA genes. Table S3. The codon usage frequency and relative synonymous codon usage (RSCU) values for the *Pedetontus silvestrii* XY protein-coding gene. Codon frequency indicates the usage frequency of each codon for encoding a specific amino acid, while the RSCU value standardizes these frequencies relative to random expectations. Table S4. The codon usage frequency and relative synonymous codon usage (RSCU) values for the *Pedetontus silvestrii* CD protein-coding gene. Codon frequency indicates the usage frequency of each codon for encoding a specific amino acid, while the RSCU value standardizes these frequencies relative to random expectations. Table S5. The codon usage frequency and relative synonymous codon usage (RSCU) values for the *Pedetontus silvestrii* DD protein-coding gene. Codon frequency indicates the usage frequency of each codon for encoding a specific amino acid, while the RSCU value standardizes these frequencies relative to random expectations. Table S6. The codon usage frequency and relative synonymous codon usage (RSCU) values for the *Pedetontus silvestrii* FC protein-coding gene. Codon frequency indicates the usage frequency of each codon for encoding a specific amino acid, while the RSCU value standardizes these frequencies relative to random expectations. Table S7. The codon usage frequency and relative synonymous codon usage (RSCU) values for the *Pedetontus silvestrii* GCL protein-coding gene. Codon frequency indicates the usage frequency of each codon for encoding a specific amino acid, while the RSCU value standardizes these frequencies relative to random expectations. Table S8. The codon usage frequency and relative synonymous codon usage (RSCU) values for the *Pedetontus silvestrii* TH protein-coding gene. Codon frequency indicates the usage frequency of each codon for encoding a specific amino acid, while the RSCU value standardizes these frequencies relative to random expectations. Table S9. Pairwise genetic distance values among *Pedetontus silvestrii* groups. The genetic distance value represents a quantitative measure of the nucleotide differences among mitochondrial loci. A higher genetic distance value indicates greater genetic differences among groups, suggesting that the groups have undergone longer periods of evolutionary separation.

## References

[1] Sturm H., Bach D.R.C. (1993). On the systematics of the Archaeognatha (Insecta). Entomol. Gen..

[2] Zhang W., Li H., Shih C., Zhang A., Ren D. (2018). Phylogenetic analyses with four new Cretaceous bristletails reveal inter-relationships of Archaeognatha and Gondwana origin of Meinertellidae. Cladistics.

[3] Grimaldi D.A. (2010). 400 million years on six legs: On the origin and early evolution of Hexapoda. Arthropod Struct. Dev..

[4] Tremmel M., Müller C. (2013). Insect personality depends on environmental conditions. Behav. Ecol..

[5] Ma Y., He K., Yu P.P., Yu D.N., Cheng X.F., Zhang J.Y. (2015). The complete mitochondrial genomes of three bristletails (Insecta: Archaeognatha): The paraphyly of Machilidae and insights into Archaeognathan phylogeny. PLoS ONE.

[6] He K., Zhang J.Y., Deng K.Z., Chen Z. (2013). The complete mitochondrial genome of the bristletail *Songmachilis xinxiangensis* (Archaeognatha: Machilidae). Mitochondr. DNA.

[7] Łukasik P., Chong R.A., Nazario K., Matsuura Y., Bublitz D.A.C., Campbell M.A., Meyer M.C., Van Leuven J.T., Pessacq P., Veloso C. (2019). One hundred mitochondrial genomes of cicadas. J. Hered..

[8] Clary D.O., Wolstenholme D.R. (1985). The mitochondrial DNA molecule of *Drosophila yakuba*: Nucleotide sequence, gene organization, and genetic code. J. Mol. Evol..

[9] Guo Z.Q., Shen C.Y., Cheng H.Y., Chen Y.X., Wu H.Y., Storey K.B., Yu D.N., Zhang J.Y. (2024). Mitogenome-based phylogeny with divergence time estimates revealed the presence of cryptic species within Heptageniidae (Insecta, Ephemeroptera). Insects.

[10] Boore J.L. (2006). The use of genome-level characters for phylogenetic reconstruction. Trends Ecol. Evol..

[11] Tong Y., Shen C.Y., Zhao Y.Y., Lin Y.J., Wu L., Storey K.B., Yu D.N., Zhang J.Y. (2022). The genetic diversity and the divergence time in extant primitive mayfly, *Siphluriscus chinensis* Ulmer, 1920 using the mitochondrial genome. Genes.

[12] Song N., Zhang H., Zhao T. (2019). Insights into the phylogeny of Hemiptera from increased mitogenomic taxon sampling. Mol. Phylogenet. Evol..

[13] Sweet A.D., Johnson K.P., Cao Y., de Moya R.S., Skinner R.K., Tan M., Herrera S.V., Cameron S.L. (2021). Structure, gene order, and nucleotide composition of mitochondrial genomes in parasitic lice from Amblycera. Gene.

[14] Hotaling S., Sproul J.S., Heckenhauer J., Powell A., Larracuente A.M., Pauls S.U., Kelley J.L., Frandsen P.B. (2021). Long reads are revolutionizing 20 years of insect genome sequencing. Genome Biol. Evol..

[15] Cameron S.L. (2024). Insect mitochondrial genomics: A decade of progress. Annu. Rev. Entomol..

[16] Linard B., Crampton-Platt A., Gillett C.P., Timmermans M.J., Vogler A.P. (2015). Metagenome skimming of insect specimen pools: Potential for comparative genomics. Genome Biol. Evol..

[17] Zhang J.Y., Zhou K.Y. (2011). Descriptions of one new genus and six new species of Machilidae (Insecta: Archaeognatha) from China: Morphological and molecular data. J. Nat. Hist..

[18] Mendes L. (1991). New contribution towards the knowledge of the Northern Korean thysanurans (Microcoryphia and Zygentoma: Insecta). Garcia Orta.

[19] Notario-Muñoz M.J., de Roca C.B., Gaju-Ricart M. (2000). *Machilinus costai*, a new species of Meinertellidae (Insecta, Microcoryphia) from Spain. Pedobiologia.

[20] Remington C.L. (1954). The suprageneric classification of the order Thysanura (Insecta). Ann. Entomol. Soc. Am..

[21] Gaju-Ricart M., Baltanás R.M., de Roca C.B. (2015). Forward without wings: Current progress and future perspectives in the study of Microcoryphia and Zygentoma. Soil Org..

[22] Giribet G., Edgecombe G.D., Carpenter J.M., D’Haese C.A., Wheeler W.C. (2004). Is *Ellipura* monophyletic? A combined analysis of basal hexapod relationships with emphasis on the origin of insects. Org. Divers. Evol..

[23] Dell’Ampio E., Szucsich N.U., Carapelli A., Frati F., Steiner G., Steinacher A., Pass G. (2009). Testing for misleading effects in the phylogenetic reconstruction of ancient lineages of hexapods: Influence of character dependence and character choice in analyses of 28S rRNA sequences. Zool. Scr..

[24] Bechly G., Stockar R. (2011). The first Mesozoic record of the extinct apterygote insect genus *Dasyleptus* (Insecta: Archaeognatha: Monura: Dasyleptidae) from the Triassic of Monte San Giorgio (Switzerland). Palaeodiversity.

[25] Korshunova T., Picton B., Furfaro G., Mariottini P., Pontes M., Prkić J., Fletcher K., Malmberg K., Lundin K., Martynov A. (2019). Multilevel fine-scale diversity challenges the ‘cryptic species’ concept. Sci. Rep..

[26] Tong Y., Wu L., Ayivi S.P.G., Storey K.B., Ma Y., Yu D.N., Zhang J.Y. (2022). Cryptic species exist in *Vietnamella sinensis* Hsu, 1936 (Insecta: Ephemeroptera) from studies of complete mitochondrial genomes. Insects.

[27] Yang Y.M., Zhang L.H., Lin Y.J., Zheng Y.M., Jin W.T., Storey K.B., Yu D.N., Zhang J.Y. (2022). The genetic diversity in *Thereuonema tuberculata* (Wood, 1862) (Scutigeromorpha: Scutigeridae) and the phylogenetic relationship of Scutigeromorpha using the mitochondrial genome. Insects.

[28] Ge J.J., Ying H.F., Xu S.Q., Huang H.T. (2023). Mitochondrial genome phylogeny reveals the deep-time origin of Gomphomastacinae (Orthoptera: Eumastacidae) and its alpine genera in China. J. Syst. Evol..

[29] Pakrashi A., Kumar V., Stanford-Beale D.A., Cameron S.L., Tyagi K. (2022). Gene arrangement, phylogeny and divergence time estimation of mitogenomes in Thrips. Mol. Biol. Rep..

[30] Wang L., Ding S., Cameron S.L., Li X., Liu Y., Yao G., Yang D. (2022). Middle Jurassic origin in India: A new look at evolution of Vermileonidae and time-scaled relationships of lower brachyceran flies. Zool. J. Linn. Soc..

[31] Shear W.A., Bonamo P.M., Grierson J.D., Rolfe W.I., Smith E.L., Norton R.A. (1984). Early land animals in North America: Evidence from Devonian age arthropods from Gilboa, New York. Science.

[32] Labandeira C.C., Beall B.S., Hueber F.M. (1988). Early insect diversification: Evidence from a Lower Devonian bristletail from Québec. Science.

[33] Grimaldi D. (2001). Insect evolutionary history from Handlirsch to Hennig, and beyond. J. Paleontol..

[34] Getty P.R., Sproule R., Wagner D.L., Bush A.M. (2013). Variation in wingless insect trace fossils: Insights from neoichnology and the Pennsylvanian of Massachussetts. Palaios.

[35] Sturm H., Poinar G.O. (1998). *Cretaceomachilis libanensis*, the oldest known bristle-tail of the family Meinertellidae (Machiloidea, Archaeognatha, Insecta) from the Lebanese Amber. Deut. Entomol. Z..

[36] Grimaldi D.A., Engel M.S., Nascimbene P.C. (2002). Fossiliferous Cretaceous amber from Myanmar (Burma): Its rediscovery, biotic diversity, and paleontological significance. Am. Mus. Novit..

[37] Mendes L.F., Wunderlich J. (2013). New data on thysanurans preserved in Burmese amber (Microcoryphia and Zygentoma lnsecta). Soil Org..

[38] Sturm H., Poinar J.G. (1997). A new *Neomachilellus* species from Miocene amber of the Dominican Republic and its phylogenetic relationships (Archaeognatha: Meinertellidae). Entomol. Gen..

[39] Riquelme F., Montejo-Cruz M., Luna-Castro B., Zuñiga-Mijangos L. (2015). Fossil jumping-bristletail from the Chiapas amber: *Neomachilellus (Praeneomachilellus) ezetaelenensis sp. nov.*(Microcoryphia: Meinertellidae). Neues Jahrb. Fur Geol. Palaontol.-Abh..

[40] Montagna M. (2020). Comment on phylogenetic analyses with four new Cretaceous bristletails reveal inter-relationships of Archaeognatha and Gondwana origin of Meinertellidae. Cladistics.

[41] Osozawa S., Nel A. (2024). Paleopteran molecular clock: Time drift and recent acceleration. Ecol. Evol..

[42] Selivon D., Perondini A.L.P., Hernandez-Ortiz V., doVal F.C., Camacho A., Gomes F.R., Prezotto L.F. (2022). Genetical, morphological, behavioral, and ecological traits support the existence of three Brazilian species of the Anastrepha fraterculus complex of cryptic species. Front. Ecol. Evol..

[43] Vrijenhoek R. (1994). DNA primers for amplification of mitochondrial cytochrome c oxidase subunit I from diverse metazoan invertebrates. Mol. Mar. Biol. Biotechnol..

[44] Zhang J.Y., Zhou C.F., Gai Y.H., Song D.X., Zhou K.Y. (2008). The complete mitochondrial genome of *Parafronurus youi* (Insecta: Ephemeroptera) and phylogenetic position of the Ephemeroptera. Gene.

[45] Burland T.G. (1999). DNASTAR’s Lasergene sequence analysis software. Bioinform. Methods Protoc..

[46] Bernt M., Donath A., Jühling F., Externbrink F., Florentz C., Fritzsch G., Pütz J., Middendorf M., Stadler P.F. (2013). MITOS: Improved de novo metazoan mitochondrial genome annotation. Mol. Phylogenet. Evol..

[47] Donath A., Jühling F., Al-Arab M., Bernhart S.H., Reinhardt F., Stadler P.F., Middendorf M., Bernt M. (2019). Improved annotation of protein-coding genes boundaries in metazoan mitochondrial genomes. Nucleic Acids Res..

[48] Tamura K., Stecher G., Kumar S. (2021). MEGA11: Molecular evolutionary genetics analysis version 11. Mol. Biol. Evol..

[49] Zhang D., Gao F.L., Jakovlić I., Zou H., Zhang J., Li W.X., Wang G.T. (2020). PhyloSuite: An integrated and scalable desktop platform for streamlined molecular sequence data management and evolutionary phylogenetics studies. Mol. Ecol. Resour..

[50] Zhang Z.Y., Guan J.Y., Cao Y.R., Dai X.Y., Storey K.B., Yu D.N., Zhang J.Y. (2021). Mitogenome analysis of four Lamiinae species (Coleoptera: Cerambycidae) and gene expression responses by *Monochamus alternatus* when infected with the parasitic nematode, *Bursaphelenchus mucronatus*. Insects.

[51] Song H., Sheffield N.C., Cameron S.L., Miller K.B., Whiting M.F. (2010). When phylogenetic assumptions are violated: Base compositional heterogeneity and among-site rate variation in beetle mitochondrial phylogenomics. Syst. Entomol..

[52] Grant J.R., Stothard P. (2008). The CGView Server: A comparative genomics tool for circular genomes. Nucleic Acids Res..

[53] Illustrator A. (2021). Adobe Illustrator. https://adobe.hmzsya.com.

[54] Perna N.T., Kocher T.D. (1995). Patterns of nucleotide composition at fourfold degenerate sites of animal mitochondrial genomes. J. Mol. Evol..

[55] Cameron S.L., Miller K.B., D’Haese C.A., Whiting M.F., Barker S.C. (2004). Mitochondrial genome data alone are not enough to unambiguously resolve the relationships of Entognatha, Insecta and Crustacea sensu lato (Arthropoda). Cladistics.

[56] Carapelli A., Liò P., Nardi F., Van der Wath E., Frati F. (2007). Phylogenetic analysis of mitochondrial protein coding genes confirms the reciprocal paraphyly of Hexapoda and Crustacea. BMC Evol. Biol..

[57] Podsiadlowski L. (2006). The mitochondrial genome of the bristletail Petrobius brevistylis (Archaeognatha: Machilidae). Insect Mol. Biol..

[58] Shen S.Q., Cai Y.Y., Xu K.K., Chen Q.P., Cao S.S., Yu D.N., Zhang J.Y. (2020). The complete mitochondrial genome of *Pedetontus zhejiangensis* (Microcoryphia: Machilidae) and its phylogeny. Mitochondr. DNA B.

[59] Guan J.Y., Shen S.Q., Zhang Z.Y., Xu X.D., Storey K.B., Yu D.N., Zhang J.Y. (2021). Comparative mitogenomes of two *Coreamachilis* species (Microcoryphia: Machilidae) along with phylogenetic analyses of Microcoryphia. Insects.

[60] Cook C.E., Yue Q., Akam M. (2005). Mitochondrial genomes suggest that hexapods and crustaceans are mutually paraphyletic. Proc. R. Soc. B Biol. Sci..

[61] Katoh K., Standley D.M. (2013). MAFFT multiple sequence alignment software version 7: Improvements in performance and usability. Mol. Biol. Evol..

[62] Castresana J. (2000). Selection of conserved blocks from multiple alignments for their use in phylogenetic analysis. Mol. Biol. Evol..

[63] Schrago C.G., Aguiar B.O., Mello B. (2018). Comparative evaluation of maximum parsimony and Bayesian phylogenetic reconstruction using empirical morphological data. J. Evol. Biol..

[64] Xia X., Xie Z. (2001). DAMBE: Software package for data analysis in molecular biology and evolution. J. Hered..

[65] Lanfear R., Calcott B., Ho S.Y., Guindon S. (2012). PartitionFinder: Combined selection of partitioning schemes and substitution models for phylogenetic analyses. Mol. Biol. Evol..

[66] Minh B.Q., Schmidt H.A., Chernomor O., Schrempf D., Woodhams M.D., Von Haeseler A., Lanfear R. (2020). IQ-TREE 2: New models and efficient methods for phylogenetic inference in the genomic era. Mol. Biol. Evol..

[67] Ronquist F., Teslenko M., Van Der Mark P., Ayres D.L., Darling A., Höhna S., Larget B., Liu L., Suchard M.A., Huelsenbeck J.P. (2012). MrBayes 3.2: Efficient Bayesian phylogenetic inference and model choice across a large model space. Syst. Biol..

[68] FigTree Version 1.4.0. http://tree.bio.ed.ac.uk/software/figtree/.

[69] Zhang J.J., Kapli P., Pavlidis P., Stamatakis A. (2013). A general species delimitation method with applications to phylogenetic placements. Bioinformatics.

[70] Yang Z., Rannala B. (2006). Bayesian estimation of species divergence times under a molecular clock using multiple fossil calibrations with soft bounds. Mol. Biol. Evol..

[71] Ho S.Y., Phillips M.J. (2009). Accounting for calibration uncertainty in phylogenetic estimation of evolutionary divergence times. Syst. Biol..

[72] Püschel H.P., O’Reilly J.E., Pisani D., Donoghue P.C. (2020). The impact of fossil stratigraphic ranges on tip-calibration, and the accuracy and precision of divergence time estimates. Palaeontology.

[73] Misof B., Liu S., Meusemann K., Peters R.S., Donath A., Mayer C., Frandsen P.B., Ware J., Flouri T., Beutel R.G. (2014). Phylogenomics resolves the timing and pattern of insect evolution. Science.

[74] Rasnitsyn A.P. (1999). Taxonomy and morphology of *Dasyleptus Brongniart*, 1885, with description of a new species (Insecta: Machilida: Dasyleptidae). Russ. Entomol. J..

[75] Keilbach R. (1982). Bibliographie und Liste der Arten tierischer Einschlüsse in fossilen Harzen sowie ihrer Aufbewahrungsorte. Dtsch. Entomol. Z..

[76] Montagna M., Haug J.T., Strada L., Haug C., Felber M., Tintori A. (2017). Central nervous system and muscular bundles preserved in a 240 million year old giant bristletail (Archaeognatha: Machilidae). Sci. Rep..

[77] Yang Z. (2007). PAML 4: Phylogenetic analysis by maximum likelihood. Mol. Biol. Evol..

[78] Rambaut A., Drummond A.J., Xie D., Baele G., Suchard M.A. (2018). Posterior summarization in Bayesian phylogenetics using Tracer 1.7. Syst. Biol..

[79] Drummond A.J., Rambaut A. (2007). BEAST: Bayesian evolutionary analysis by sampling trees. BMC Evol. Biol..

[80] Jungreis I., Chan C.S., Waterhouse R.M., Fields G., Lin M.F., Kellis M. (2016). Evolutionary dynamics of abundant stop codon readthrough. Mol. Biol. Evol..

[81] Bachtrog D., Thornton K., Clark A., Andolfatto P. (2006). Extensive introgression of mitochondrial DNA relative to nuclear genes in the *Drosophila yakuba* species group. Evolution.

[82] Yuan Y., Kong L., Li Q. (2013). Mitogenome evidence for the existence of cryptic species in *Coelomactra antiquata*. Genes Genom..

[83] Powell J.R., Moriyama E.N. (1997). Evolution of codon usage bias in *Drosophila*. Proc. Natl. Acad. Sci. USA.

[84] Rao Y.S., Wu G.Z., Wang Z.F., Chai X.W., Nie Q.H., Zhang X.Q. (2011). Mutation bias is the driving force of codon usage in the *Gallus gallus* genome. DNA Res..

[85] Mandal G. (2023). Fauna of India Checklist: Arthropoda: Insecta: Archaeognatha. Version.

[86] DiMichele W.A., Pfefferkorn H.W., Gastaldo R.A. (2001). Response of Late Carboniferous and Early Permian plant communities to climate change. Annu. Rev. Earth Planet. Sci..

[87] Chaloner W.G. (1967). Spores and land-plant evolution. Rev. Palaeobot. Palynol..

[88] Cleal C.J., Uhl D., Cascales-Miñana B., Thomas B.A., Bashforth A.R., King S.C., Zodrow E.L. (2012). Plant biodiversity changes in Carboniferous tropical wetlands. Earth-Sci. Rev..

[89] Lucas S.G., DiMichele W.A., Opluštil S., Wang X. (2023). An Introduction to Ice Ages, Climate Dynamics and Biotic Events: The Late Pennsylvanian World. https://www.lyellcollection.org/doi/full/10.1144/SP535-2022-334.

[90] Zachos J., Pagani M., Sloan L., Thomas E., Billups K. (2001). Trends, rhythms, and aberrations in global climate 65 Ma to present. Science.

[91] Witts J.D., Newton R.J., Mills B.J., Wignall P.B., Bottrell S.H., Hall J.L., Francis J.E., Crame J.A. (2018). The impact of the Cretaceous–Paleogene (K–Pg) mass extinction event on the global sulfur cycle: Evidence from Seymour Island, Antarctica. Geochim. Cosmochim. Acta.

[92] Bateman R.M. (2020). Hunting the Snark: The flawed search for mythical Jurassic angiosperms. J. Exp. Bot..

[93] Givnish T.J. (2015). Adaptive radiation versus ‘radiation’ and ‘explosive diversification’: Why conceptual distinctions are fundamental to understanding evolution. New Phytol..

[94] Feldberg K., Heinrichs J., Schmidt A.R., Váňa J., Schneider H. (2013). Exploring the impact of fossil constraints on the divergence time estimates of derived liverworts. Plant Syst. Evol..

[95] Traverse A. (1982). Response of world vegetation to Neogene tectonic and climatic events. Alcheringa.

[96] Laenen B., Shaw B., Schneider H., Goffinet B., Paradis E., Désamoré A., Heinrichs J., Villarreal J., Gradstein S., McDaniel S. (2014). Extant diversity of bryophytes emerged from successive post-Mesozoic diversification bursts. Nat. Commun..

[97] Bippus A.C., Flores J.R., Hyvönen J., Tomescu A.M. (2022). The role of paleontological data in bryophyte systematics. J. Exp. Bot..

[98] Duan M., Tu J., Lu Z. (2018). Recent advances in detecting mitochondrial DNA heteroplasmic variations. Molecules.

[99] Ponti R., Sannolo M. (2023). The importance of including phenology when modelling species ecological niche. Ecography.

