# Supplementary material for: Morpho-Molecular Discordance and Cryptic Diversity in Jumping Bristletails: A Mitogenomic Analysis of Pedetontus silvestrii (Insecta: Archaeognatha: Machilidae)
Source: Insects. 2025 Apr 25;16(5):452. doi: 10.3390/insects16050452 (PMC12112178; doi:10.3390/insects16050452)

Figure S2. The results from the Bayesian phylogenetic species delimitation analysis (bPTP) server. The bPTP analysis determines the number of different species by evaluating genetic differences and phylogenetic relationships. This figure includes the posterior probabilities supporting species boundaries and the hierarchical structure of species clusters.

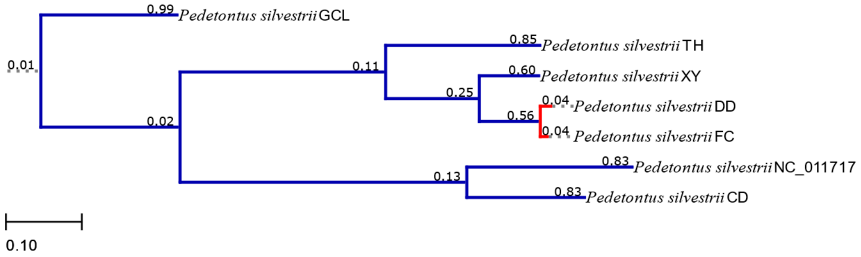

Supplement: Supplementary file 1 [file insects-16-00452-s001.zip › Figure S2.pdf]
