# Supplementary material for: Morpho-Molecular Discordance and Cryptic Diversity in Jumping Bristletails: A Mitogenomic Analysis of Pedetontus silvestrii (Insecta: Archaeognatha: Machilidae)
Source: Insects. 2025 Apr 25;16(5):452. doi: 10.3390/insects16050452 (PMC12112178; doi:10.3390/insects16050452)

Figure S3. Synonymous codon usage bias in the *Pedetontus silvestrii* mitogenome was systematically evaluated through codon preference analysis. The visualization framework employs codon variants mapped along the abscissa and RSCU measurements plotted on the ordinate. This metric quantifies codon preference by measuring observed-to-expected usage ratios across translational coding se-quences.

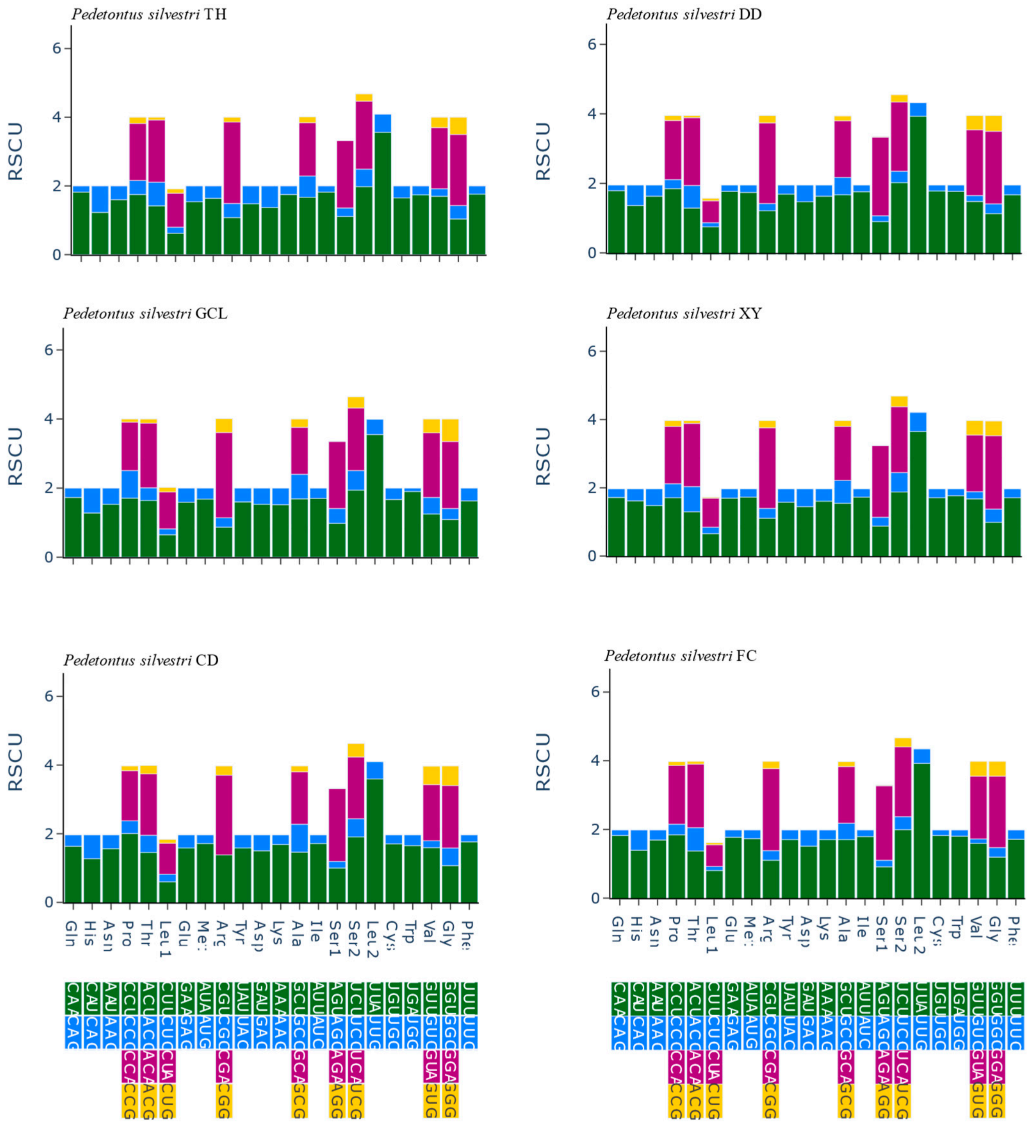

Supplement: Supplementary file 1 [file insects-16-00452-s001.zip › Figure S3.pdf]
