# Supplementary material for: Morpho-Molecular Discordance and Cryptic Diversity in Jumping Bristletails: A Mitogenomic Analysis of Pedetontus silvestrii (Insecta: Archaeognatha: Machilidae)
Source: Insects. 2025 Apr 25;16(5):452. doi: 10.3390/insects16050452 (PMC12112178; doi:10.3390/insects16050452)
Supplement: Supplementary file 1 [file insects-16-00452-s001.zip › Table S9.pdf]

Table S9 Pairwise genetic distance values among *Pedetontus silvestrii* groups. The genetic distance value represents a quantitative measure of the nucleotide differences among mitochondrial loci. A higher genetic distance value indicates greater genetic differences among groups, suggesting that the groups have undergone longer periods of evolutionary separation.

	P. silvestrii DD<br>*P. silvestrii* FC<br>*P. silvestrii* XY	*P. silvestrii* GCL	*P. silvestrii* TH	*P. silvestrii* CD	*P. silvestrii* (NC_011717)
P. silvestrii DD<br>*P. silvestrii* FC<br>*P. silvestrii* XY					
P. silvestrii GCL	0.173				
P. silvestrii TH	0.116	0.178			
P. silvestrii CD	0.183	0.181	0.194		
P. silvestrii (NC_011717)	0.186	0.185	0.195	0.107	
