# Supplementary material for: Morpho-Molecular Discordance and Cryptic Diversity in Jumping Bristletails: A Mitogenomic Analysis of Pedetontus silvestrii (Insecta: Archaeognatha: Machilidae)
Source: Insects. 2025 Apr 25;16(5):452. doi: 10.3390/insects16050452 (PMC12112178; doi:10.3390/insects16050452)

**Figure S1.** Circular genome maps illustrate the complete mitochondrial genomes of six P. silvestrii variants: DD, FC, GCL, TH, CD, and XY. Strand-specific coding is represented through concentric circles, with the outer circle (positive strand/5'→3') contrasting with the inner circle (negative strand/3'→5'). Subsequent rings depict: (1) GC-skew deviations, and (2) variations in GC content relative to genomic averages.

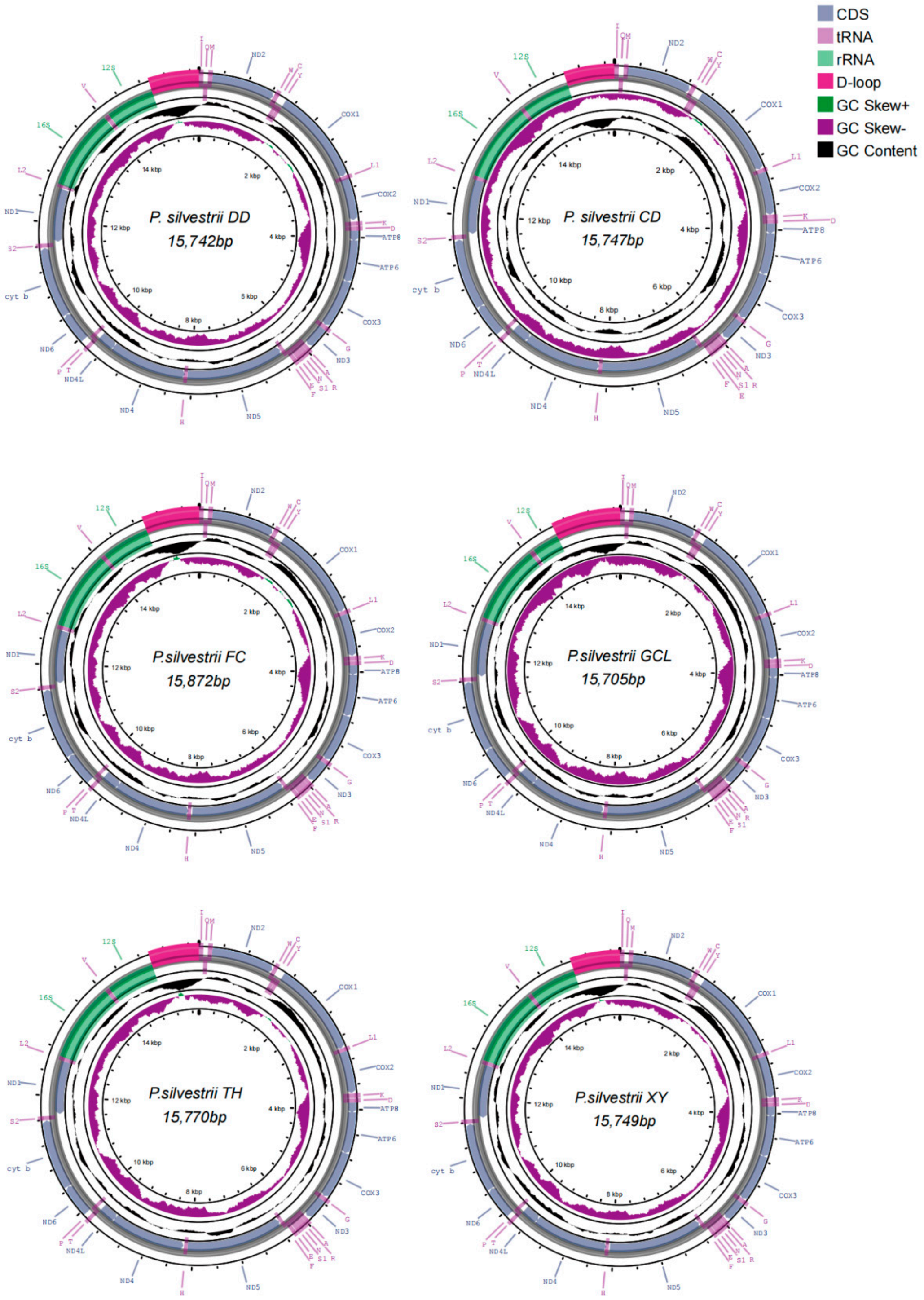

Supplement: Supplementary file 1 [file Figure S1.pdf › Figure S1.pdf]
